# Xenopus LAP2β protein knockdown affects location of lamin B and nucleoporins and has effect on assembly of cell nucleus and cell viability

**DOI:** 10.1007/s00709-015-0861-y

**Published:** 2015-07-25

**Authors:** Magda Dubińska-Magiera, Magdalena Chmielewska, Katarzyna Kozioł, Magdalena Machowska, Christopher J. Hutchison, Martin W. Goldberg, Ryszard Rzepecki

**Affiliations:** 1 Laboratory of Nuclear Proteins, Faculty of Biotechnology, University of Wrocław, Joliot- Curie 14a, 50-383 Wrocław, Poland; 2 Department of Animal Developmental Biology, Institute of Experimental Biology, University of Wroclaw, Sienkiewicza 21, 50-335 Wroclaw, Poland; 3 Department of Evolutionary Biology and Vertebrate Conservation, University of Wroclaw, Sienkiewicza 21, 50-335 Wroclaw, Poland; 4 Integrative Cell Biology Laboratory, School of Biological and Biomedical Sciences, The University of Durham, South Road, Durham, DH1 3LE UK

**Keywords:** LAP2, Knockdown, Cell nucleus, Nucleoporin, Lamin B, TPX2, *Xenopus laevis*

## Abstract

Xenopus LAP2β protein is the single isoform expressed in XTC cells. The protein localizes on heterochromatin clusters both at the nuclear envelope and inside a cell nucleus. The majority of XLAP2β fraction neither colocalizes with TPX2 protein during interphase nor can be immunoprecipitated with XLAP2β antibody. Knockdown of the XLAP2β protein expression in XTC cells by synthetic siRNA and plasmid encoded siRNA resulted in nuclear abnormalities including changes in shape of nuclei, abnormal chromatin structure, loss of nuclear envelope, mislocalization of integral membrane proteins of INM such as lamin B2, mislocalization of nucleoporins, and cell death. Based on timing of cell death, we suggest mechanism associated with nucleus reassembly or with entry into mitosis. This confirms that Xenopus LAP2 protein is essential for the maintenance of cell nucleus integrity and the process of its reassembly after mitosis.

**Electronic supplementary material:**

The online version of this article (doi:10.1007/s00709-015-0861-y) contains supplementary material, which is available to authorized users.

## Introduction

Lamina-associated polypeptide 2 (LAP2) proteins are alternatively spliced proteins belonging to the family of LEM domain proteins (for review, see (Schirmer and Foisner 2007, Dorner et al. 2007, Wagner and Krohne 2007). The LEM domain family in humans was originally composed of three proteins (LAP2, emerin, and MAN1). Since then, four additional LEM domain proteins have been discovered in mammals. These are NET-25 (Lem2), Lem3, Lem4, and Lem5 (Lee and Wilson 2004, Berk et al. 2013). Mutations in genes coding for proteins of the nuclear lamina (also members of a LEM-domain family) lead to laminopathies, a group of rare genetic disorders (for review, see Zaremba-Czogalla et al. 2011a, Dubinska-Magiera et al. 2013).

LAP2 proteins are expressed in metazoans, excluding *Caenorhabditis elegans* but including *Drosophila melanogaster*, *Danio rerio*, *Xenopus laevis*, *Mus musculus*, and *Homo sapiens*. LAP2 proteins are alternatively spliced products of a single gene, resulting in integral membrane or nucleoplasmic (or rarely cytoplasmic) proteins (Harris et al. 1994). Their unique feature is the similar conservative structure, especially among vertebrate orthologues. LAP2 proteins comprise N-terminal “common” domain containing LEM domain and LEM-like domain, both interacting with barrier to autointegration factor (BAF) (Furukawa 1999, Shumaker et al. 2001) or DNA (chromatin) (Cai et al. 2001). The rest of the particular LAP2 protein structure and function depends on what exons were incorporated into mature mRNA during alternative splicing of the primary transcript. The “variable” domain typically contains lamin binding domain or specifically lamin B-binding domain and domains responsible for binding to germ-cell-less (GCL) (Nili et al. 2001), HA95 protein (Martins et al. 2003), and histone deacetylase 3 (HDAC3) protein (Somech et al. 2005). Alternative splicing also generates protein isoforms with or without a transmembrane domain. This results in an appearance of integral membrane or “soluble” protein isoform. In mammals, six LAP2 protein isoforms have been discovered (α, β, γ, δ, ε, and ζ). LAP2α and LAP2 ζ proteins lack transmembrane domains and are soluble nucleoplasmic and cytoplasmic proteins, respectively (Shaklai et al. 2008). The most thoroughly studied isoform was mammalian LAP2α protein due to its ability to modulate signaling pathway associated with Rb protein (LAP2α, lamin A, pRb complex) and regulate gene transcription associated inter alia with cell cycle progression into S-phase (Gant et al. 1999, Markiewicz et al. 2002, Yang et al. 1997, Pekovic et al. 2007). Mammalian LAP2β isoforms are also involved in general gene regulation and as transcriptional repressors (Somech et al. 2005). They play a crucial role in attachment of chromatin to the nuclear envelope (NE) and nuclear lamina (NL) during interphase and nuclear reassembly after mitosis.

In *X. laevis*, there have been five cDNA sequences identified that are presumably translated into three different LAP2 proteins discovered so far, ω, β, and γ (Gant et al. 1999, Lang et al. 1999, Lang and Krohne 2003, Chmielewska et al. 2011). The Xenopus LAP2β (66 kDa somatic polypeptide) is the only one found in adult animals, whereas two other isoforms, ω and γ (86 and 40 kDa), are present in oocytes and eggs and are downregulated during embryogenesis (Chmielewska et al. 2011). The N-terminal common fragment of XLAP2, when added to the in vitro nuclear assembly reaction, inhibits chromatin decondensation and nuclear growth similarly to the human LAP2β N-terminal fragment (Gant et al. 1999). Moreover, XLAP2 β (66 kDa polypeptide) coprecipitates with lamin B2 and A from adult Xenopus tissues and A6 cells (Lang and Krohne 2003). The N-terminal domain of XLAP2 (aa 1-165), like other vertebrate LAPs, interacts with BAF and BAF-DNA complexes. The in vitro interaction of XLAP2 protein isoforms from *X. laevis* egg extracts—XLAP2ω and XLAP2γ with a spindle assembly factor—TPX2 (targeting protein for Xklp2) was confirmed. XLAP2-TPX2 complex is therefore thought to be required for proper assembly of postmitotic nuclei in *X. laevis* in vitro nuclear assembly system (O'Brien and Wiese 2006).

Recently, we confirmed the presence of at least three XLAP2 isoforms, ω, β, and γ, that were developmentally regulated (Chmielewska et al. 2011). XLAP2 proteins colocalize with lamin B2 and B3 during development and lamin B2 in adult tissues. We also demonstrated that Xenopus LAP2β localizes both at the NE and inside the nucleus in clusters of heterochromatin. The intranuclear clusters of XLAP2β on heterochromatin were found to be partly independent of NE invaginations. We also demonstrated that in XTC cells, LAP2β is the sole LAP2 isoform expressed.

In this study, we examined the effect of knockdown of LAP2β protein synthesis in *X. laevis* XTC cells and its effect on cell viability and cell nucleus structure and function.

## Materials and methods

### Plasmids, cDNAs and siRNAs, and tissue culture

Sequences of siRNAs for XLAP2 knockdown were designed using the online Invitrogen tool [https://rnaidesigner.invitrogen.com/rnaiexpress/?CID=TN-Tools-BlockiT] on the template of XLAP2 clone2 cDNA sequence, AF048815 (Gant et al. 1999): 15 XLAP2 sense strain: 5′- GCAAGACCCGUCGGUACUUACUAAA -3′, 98 XLAP2 sense strain:, 5′- GGAAAGAUGUGUAUGUGCAACUCUA-3′, and 237 XLAP2 sense strain, 5′- GAAGACCGACAAACCUAGAGCAGAA-3′. Scrambled control siRNA sequence was: control 15 (c15) sense, 3′-GCACCAUGCGGCCAUAUUUCAGAAA-5′.

*Xenopus laevis* XTC cells (Pudney et al. 1973) were grown in 54 % L-15 Leibovitz medium containing 10 % FBS, 2 mM l-glutamine, 10 I.U./ml penicillin, 10 μg/ml streptomycin, and 0.025 μg/ml amphotericin B at 22–26 °C in normal air conditions as described previously (Chmielewska et al. 2011).

Cells were transfected with 100 nM specific or control (scrambled) siRNAs using Oligofectamine reagent (Life Technologies). For plasmid-based siRNA knockdown, plasmid pFIV-H1/U6-copGFP (SBI System Bioscience, USA) with inserted sequences of control (C15) and active (15) sequences for siRNA was used. Transfection was with Metafectene Pro (Biontex, Germany) with 1 μg of plasmid DNA per 6 μl of transfection reagent. Cells were grown on 24-well plates at an initial density of 6 × 10^4^ per well. Cells were collected after 24, 48, 72, or 96 h after transfection, depending on the experiment.

### Microscope procedures

For imaging, confocal microscope LSM 510 META with FCS system was used. For imaging in non-confocal mode, fluorescence microscope (Olympus IX70) was used. Any brightness and contrast adjustments were performed in Adobe Photoshop, Zen 2007 (Zeiss) or ImageJ (Schneider et al. 2012).

### Statistical analysis

For statistical analyses of cellular phenotypes, the following procedure was used: Samples of XTC cells grown on coverslips were fixed for immunofluorescence microscopy and stained for XLAP2. Ten representative fields from each sample (three independent experiments) were imaged under ×10 objective. In the digital images, the total cell number was counted. The statistical analysis was performed using Statistica software (StatSoft, Poland). Groups of data were compared utilizing the Student’s *t* test. Statistical significance was assumed at values of *P* < 0.05. Cell phenotypes were examined in the digital images distinguished on the basis of structural criteria of shape and size of cells and nuclei, organization of microtubular network, cell adherence to the surface, degree of cell shrinking, and apoptotic phenotype (determined by abnormal chromatin condensation, the occurrence of vesicles within the nucleus, and lack of NE staining).

For calculations of number of cells with knockdown XLAP2 protein XTC cells, 48 h after transfection with plasmid-based siRNA constructs “c15” or “15”, were fixed with 4 % paraformaldehyde (PFA), stained with anti-XLAP2 antibody and DAPI, and visualized with confocal microscope. Fifty cells demonstrated GFP expression were counted for each specimen. When counting, the XLAP2 expression level and shape of nuclei were estimated also. Counting was performed for three series of transfections and averaged. Experiments on knockdown of XLAP2β protein (and controls) were always performed simultaneously, exactly at the same session on a confocal microscope. We used the same aliquots of antibodies and the same settings of the microscope. When differences in optical density of the staining were questionable, we performed optical density measurements of NE staining. As a cell with XLAP2 knockdown, we counted cells with an optical density lower than 50 % of a density of control from the same slide. Typically, values for normal NE were about 150 or higher. Random views from slides were selected for observations. For GFP fluorescence (reporter protein for transfection), the threshold was estimated at the background level increased by 100 %. Typically, background level was about 20–25 depending on the slide, which means that the threshold was set at 40 to 50 depending on the measured background of currently analyzed slides. Only cells from the same slides were compared. All calculations were made on cells fixed with PFA only since other fixation methods did not preserve the GFP in order to allow reproducible measurements (see Fig. 6).

For calculation of cells number, expressing a detectable level of TPX2 protein, exponentially growing XTC cells were fixed with 4 % PFA, stained with anti-TPX2 antibody and DAPI, and visualized with a confocal microscope. 200 cells were counted. Percentage of cells with the detectable TPX2 signal was calculated, and phases of the cell cycle for those cells were estimated using FACS analyses.

### Colocalization studies

For qualitative colocalization analysis (relative fluorescence intensities measurements), images acquisition were performed on a Zeiss LSM 510 Meta confocal microscope. Distribution of fluorescence intensities along the cross-section of the typical cell was done using “Profile” function of the Zeiss ZEN 2009 software (Carl Zeiss MicroImaging). Resulting graph shows signal intensities in accordance with chosen section (red arrow). To set threshold, point marker (cyan dot or line) was chosen. To show differences in XLAP2 and TPX2 fluorescence intensities in the nuclear envelope, the second point marker (violet dot or line) was set. The calculations for these chosen points are presented in tables.

### Quantitative colocalization analysis

For quantification of colocalization, image acquisition was performed on a Zeiss LSM 510 Meta confocal microscope. Colocalization of proteins’ signals was determined using Zeiss ZEN 2009 software (Carl Zeiss MicroImaging). The threshold was set for each pair of channels by selecting region of interest (ROI) outside the cells. Subsequently, the ROIs at the intranuclear area, at the nuclear envelope, or in the cytoplasm were chosen, and colocalizations were calculated. For each case, five ROIs, coming from separate cells, were analyzed. Finally, averaged calculations, graphs, and statistical analysis were done based on percentage of signals from lamin B2 channel that are colocalized with signals from XLAP2 and TPX2 in the analyzed ROI and is insensitive to differences in signal intensities.

### Antibodies, immunofluorescence, and Western blotting

For immunofluorescence (IF), XTC cells were grown on coverslips, fixed with 4 % PFA in phosphate buffered saline (PBS) for 15 min, permeabilized with 0.5 % Triton X-100 in PBS (*v*/*v*) for 5 min, and blocked with 1 % fetal bovine serum (FBS) in PBS (for 30 min at room temperature). Alternatively, to use selected antibodies, cells were fixed with ice-cold methanol for 10 min in −20 °C without separated permeabilization step. All wash steps were done with PBS. Primary antibodies were used for overnight incubation at 4 °C and secondary antibodies for 60 min at room temperature. DNA was stained with DAPI (4,6-diamidino-2-phenylindole, 0.2 μg/ml in mounting medium with Mowiol, and DABCO). For imaging, confocal microscope LSM 510 META with FCS system was used. Any brightness and contrast adjustments were performed in Adobe Photoshop, Zen 2007 (Zeiss) or ImageJ (Schneider et al. 2012).

The specific serum and antibodies for an N-terminal fragment of XLAP2 protein were produced as described previously (Rzepecki et al. 1998).

The following antibodies were used: rabbit affinity purified anti-N-TPX2 antibody (1:100 IF) was a kind gift from Prof. Y. Zheng (Tsai et al. 2006), antibodies anti-XBAF protein (1:50 IF) were from Prof. P.A. Fisher and Prof. K. Furukawa (Dechat et al. 2004, Furukawa et al. 2003), and affinity purified IgGs: anti-XLAP2 (1:100 WB, 1:60 IF) (Salpingidou et al. 2008, Chmielewska et al. 2011). Mouse monoclonal antibodies: anti-*X. laevis* lamin B2 ab (1:25 IF, Santa Cruz Biotechnology sc-56147), Ab 414 against nucleoporins with F/G repeats (1:100 IF, Covance MMS-120P), Ac-40 actin Ab (1:800 WB, Sigma), rat monoclonals anti-alpha-tubulin YL1/2 (1:60, Serotec), anti-β-tubulin (1:150 IF, Sigma T4026), and anti-γ-tubulin (1:100 IF, Sigma T5326). For F-actin identification, Alexa Fluor^®^ 546 phalloidin was used (at a concentration of 2 U*/*ml*;* Invitrogen). Cellular lipid membranes were stained with DHCC (3,3″-dihexyloxacarbocyanine iodide at a concentration of 7.5 μg/ml; Invitrogen). Secondary antibodies for immunoblotting and fluorescence were from Jacksons ImmunoResearch.

Proteins were separated on 10 or 12 % SDS-PAGE gels and electro-transferred onto nitrocellulose filters. For XLAP2 silencing analyses, optical density measurements of the protein bands in immunoblots were performed with the BIO-PROFIL Bio-1D Windows Application V99.01. Protein content in protein bands was normalized according to the actin content in each lane. Western blotting analysis of the XLAP2 protein silencing in XTC cells was monitored by staining with antibodies against XLAP2 and actin staining with mAb to beta actin (AC-40, SIGMA) was used as loading control. Optical density of XLAP2 and actin bands was measured with the BIO-PROFIL Bio-1D Windows Application V99.01, and XLAP2 content was normalized versus actin for each lane.

### Immunoprecipitation and mass spectroscopy

Immunoprecipitation (IP) and mass spectroscopy (MS) were performed essentially as described previously (Rzepecki et al. 1998, Chmielewska et al. 2011, Zaremba-Czogalla et al. 2011b) with minor modifications. All co-IP experiments were performed under native conditions under different ionic strength in order to extract fractions of XLAP2β protein with different solubility. Co-IP procedure number 1 was performed with extraction, binding and washing the beads with buffer containing 0.3 M NaCl. The procedure number 2 was performed with 0.6 M NaCl and procedure number 3 with 0.6 M NaCl in extraction buffer and 0.3 M NaCl during the rest of the procedure. For each co-IP procedure, we used 9 × 10^6^ cells, and for Western blot analyses, equivalents of 2 × 10^5^ cells/lane were loaded. Only proteins with “identification score” of 100 % in at least one IP procedure were shown.

### Mass spectrometry

For identification of coimmunoprecipitated proteins, tandem mass spectrometry was conducted essentially as described previously (Chmielewska et al. 2011, Zaremba-Czogalla et al. 2011b).

## Results

### Localization of XLAP2β in cultured XTC cells during interphase

In order to get an insight into the cell cycle-dependent localization of XLAP2β in comparison to other important cellular proteins, we used XTC cells that express this single isoform and have a fibroblast-like phenotype (Chmielewska et al. 2011).

**Fig. 1:**
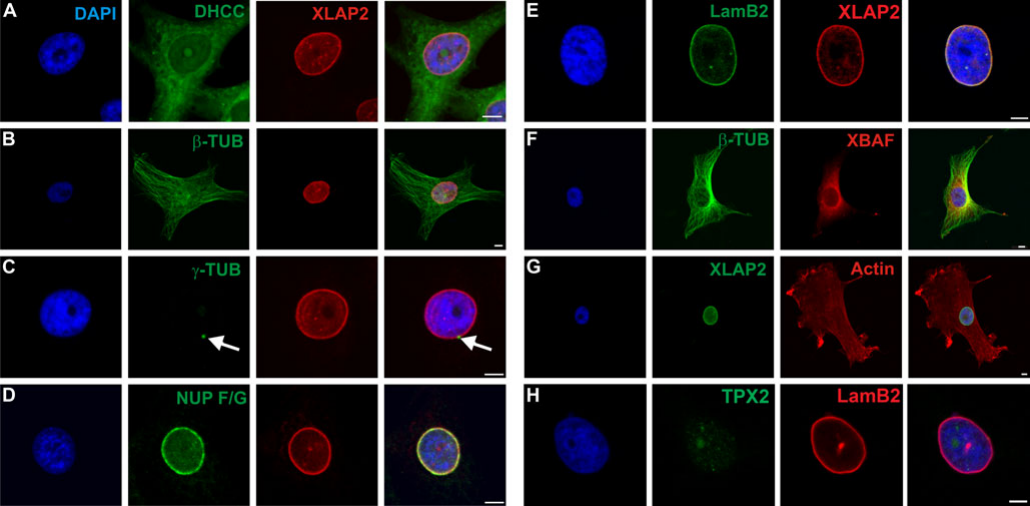
XLAP2β localizes in *X. laevis* XTC cells typically for the inner nuclear membrane protein. Cells were grown on coverslips, fixed with PFA or methanol, and stained for XLAP2β, lamin B2, nucleoporins F/G, XBAF, TPX2, actin, γ-tubulin, β-tubulin, and membranes (DHCC). DNA was visualized with DAPI (*blue*). Single confocal sections (1.5 μm) through the center of nuclei are shown. Bar: 5 μm. Interphase XLAP2β protein colocalizes with lamin B2, shows apparent colocalization with FG-repeat nucleoporins, and partly colocalizes with BAF proteins at the NE only. LAP2β does not localize to cytoplasm or ER. In XTC cells, centrosomes locate immediately next to NE. XTC cells show a fibroblast-like distribution of microtubules and actin. In interphase XTC cells, TPX2 protein is weakly expressed. It gradually appears in the mid-S phase and is dispersed through the whole nucleoplasm so does not colocalize with XLAP2β

Interphase XLAP2β protein shows typical location for integral protein of inner nuclear membrane (INM), colocalizes with lamin B2, shows apparent colocalization with FG-repeat nucleoporins, and does not localize to cytoplasm or endoplasmic reticulum (ER) (Fig. 1). Centrosomes (MTOCs) locate immediately next to NE in XTC cells. BAF protein is distributed at the NE and surrounding cytoplasm. LAP2β partly colocalizes only with BAF protein at the NE. Please note that XTC cells show a fibroblast-like distribution of microtubules and actin (Fig. 1).

**Fig. 2:**
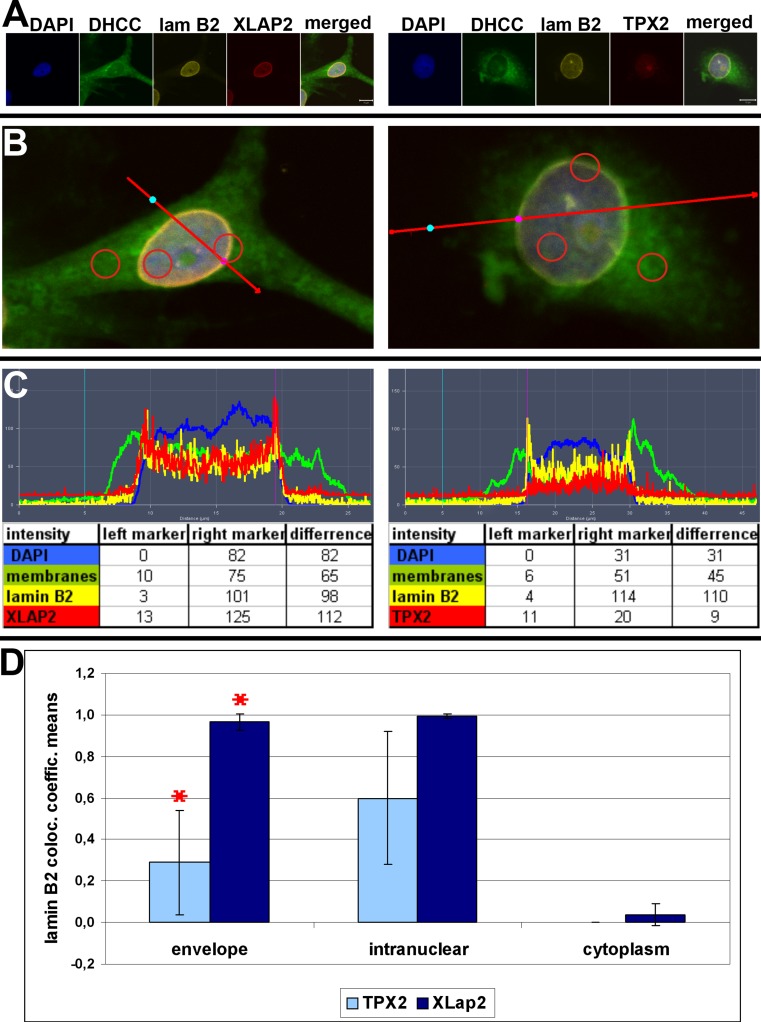
The main fraction of XLAP2β does not colocalize with TPX2 in XTC cells during interphase. XTC cells were grown on coverslips, fixed with methanol, and stained for XLAP2β, lamin B2, TPX2, and membranes (DHCC). DNA was visualized with DAPI (*blue*). Single confocal sections (1.5 μm) through the center of nuclei are shown in **a**. Bar: 10 μm. They illustrate typical localization for those antigens in XTC cells. For TPX2 localization and all analyses of its colocalization, only cells with high level (easily detectable) of TPX2 were chosen. **b** Line sections (*long red arrows*) used for qualitative colocalization analyses (relative fluorescence intensities distribution). **c** Results of these analyses. Left marker (*cyan*) indicates point outside the cell (threshold) and right marker (*violet*) points the nuclear envelope. It is clearly seen that TPX2 does not localize to nuclear envelope where the main signal from XLAP2 locates. **b** Presents three type of regions of the cell (intranuclear, envelope, and cytoplasm), shown as *red circles*, which were the basis for quantitative colocalization calculations resulted in the graph displayed in **d**. *Bars* of the chart indicate colocalization coefficients of lamin B2 with XLAP2 either TPX2. *Red asterisks* show statistically significant differences between analyzed colocalizations. Lamin B2 colocalizes with XLAP2 in the nuclear envelope and in the nuclear interior but only partially with TPX2. In the envelope, the difference between colocalization coefficients for XLAP2 and for TPX2 is statistically significant, and inside the nucleus is on the borderline of significance. Hence, TPX2 and XLAP2 do not colocalize in examined regions

We also analyzed the distribution of TPX2 protein in interphase XTC cells (Fig. 1) due to the previous report that XLAP2 ω and γ proteins interact with TPX2 protein from *Xenopus* egg extracts (O'Brien and Wiese 2006). We found that most of XTC cells demonstrated weak or no staining with anti-TPX2 antibodies (Additional file 1: Figure S1B). TPX2 protein is distributed through the nuclear interior. Detailed analyses revealed that only about 40 % of interphase cells demonstrate clearly detectable by immunofluorescence staining, higher level of TPX2 protein and that the location of the protein is intranuclear (Additional file 1: Figure S1). The analyses of the cell cycle in XTC cells using FACS method indicated that this 40 % of cells correlates roughly with a percentage of cells in S-phase together with G2/M-phase (not shown). This feature of frog TPX2 reflects the behavior of mammalian TPX2 protein (Stewart and Fang 2005). Since our XLAP2β antibody and TPX2 antibody were raised in rabbits, we could not perform direct colocalization studies between these two proteins. That is why we used indirect approach and studied XLAP2β and TPX2 protein colocalization with lamin B2 and analyzed the distribution of these proteins through the nuclear compartment. Figure 2a demonstrates typical example for staining of XTC cells for DNA, membranes, XLAP2, TPX2, and lamin B2. Figure 2b demonstrates merged images with line sections taken for fluorescence intensities analyses shown in Fig. 2c. Figure 2b also indicates a marked different type of regions used for calculation of colocalization coefficients illustrated in Fig. 2d. The demonstrated data indicate that there is no significant fraction of XLAP2β and TPX2 protein colocalizing with each other, particularly at the nuclear envelope location where, additionally, it localizes only residual fraction of TPX2. This suggests that major fractions of both proteins may not interact with each other.

In order to detect potential interactions between XLAP2 and TPX2 proteins, we used immunoprecipitation for XLAP2 followed by mass spectroscopy for identification of co-immunoprecipitated proteins from unsynchronized XTC cell extracts. The results of such experiments performed under three different ionic conditions are demonstrated in Additional file 2: Figure S2. TPX2 protein was not detected in any of the samples with XLAP2β co-immunoprecipitated proteins. Also, our previous studies on XLAP2β protein using IP and mass spectrometry did not show TPX2 as an interacting partner (Chmielewska et al. 2011) (22). Thus, our data indicate that most of Xenopus TPX2 do not colocalize with the majority of LAP2β in XTC cells during interphase. This means that XLAP2β protein (unlike XLAP2ω and γ) is not responsible for TPX2 protein retention in cell nucleus during interphase.

### siRNA mediated knockdown of XLAP2β results in nuclear and cellular abnormalities and cell death

**Fig. 3:**
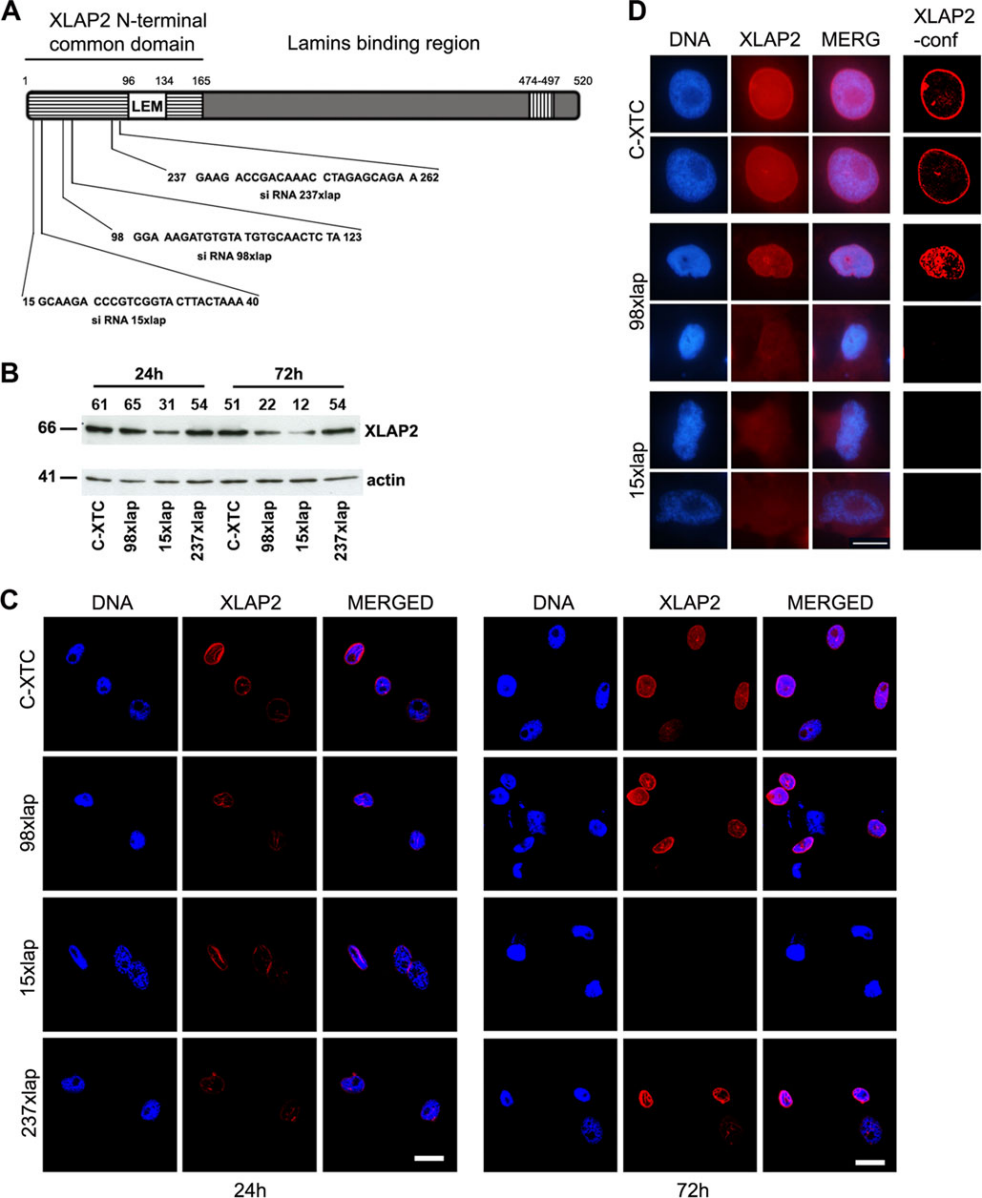
Analysis of the XLAP2 protein knockdown in tissue cultured *X. laevis* XTC cells. XTC cells were plated at 7.5 × 10^4^ cells/well, transfected with 100 nM siRNA sequences, and grown for 24 and 72 h. C-XTC, untreated cells; 15, 98, 237xlap, cells treated with different siRNAs. **a** Schematic diagram of the Xenopus LAP2β protein. The positions of amino acid residues are marked above the diagram. Locations of predicted domains are marked by different patterns: *striped box* common domain of all potential isoforms, *white box* LEM domain, *hatched box* the transmembrane region. cDNA sequences given below the diagram were used to design 25 nt ds siRNAs for XLAP2 protein expression silencing. *Numbers* denote a position of nucleotides. **b** Western blotting analysis of the XLAP2 protein silencing in tissue cultured *X. laevis* XTC cells after 24 and 72 h post- transfection. Equal amounts of the protein were loaded. *Numbers denoted above each lane* represent the relative amounts of XLAP2 protein. Molecular masses of the proteins (kDa) are marked on the left side of the picture. XLAP2 knockdown was most efficient with 98xlap and 15xlap siRNAs after 3 days post-transfection. **c**, **d** Effect of XLAP2 protein knockdown on the morphology of XTC cells was also analyzed by confocal and immunofluorescence microscopy. XTC cells were stained for XLAP2 (*red*), DNA was visualized with DAPI (*blue*). **c** General view of the control, non-treated, and siRNA transfected XTC cells. Note the complete loss of XLAP2 signal after 72 h post-transfection with 15xlap siRNA. **d** Higher magnification of the control and silenced nuclei imaged in non-confocal (3 *left columns*) or confocal mode (*right column*) visualize that the degrading XLAP2 protein is no longer localized in the NE. DNA is amorphous (98xlap). Note the apoptotic nucleus with lobulations (15xlap lower photograph). Images were combined and processed in Adobe Photoshop. Single confocal sections (1.5 μm) through the center of nuclei are shown. Bars, 20 μm (**c**), 10 μm (**d**)

In order to unveil the cellular function of LAP2β protein in *X. laevis*, we used siRNA to knockdown the expression of a single XLAP2β isoform present in XTC cells. Figure 3 shows a schematic diagram of the protein, sequences of the siRNA fragments, their location in N-terminal “common domain,” and the results of the knockdown experiment. The most efficient knockdown effect, as assayed by Western blot, showed 15xlap siRNA (80 %) and the lesser 98xlap (64 %), while 237xlap siRNA showed no effect (Fig. 3b). Similar data were obtained by IF and confocal analyses, where it was possible to observe the decreased level of XLAP2 protein (Fig. 3c, 237lap, 98lap) or its apparent disappearance after 72 h (Fig. 3c, 15xlap). Detailed analyses of the cell nuclei revealed almost complete loss of the XLAP2 protein from the NE in cells treated with 15xlap siRNA. This was associated with the nuclear shape abnormalities (Fig. 3d), similar to lamin A/C knockout fibroblasts or EDMD (Emery-Dreifuss muscular dystrophy) patients (Sullivan et al. 1999).

**Fig. 4:**
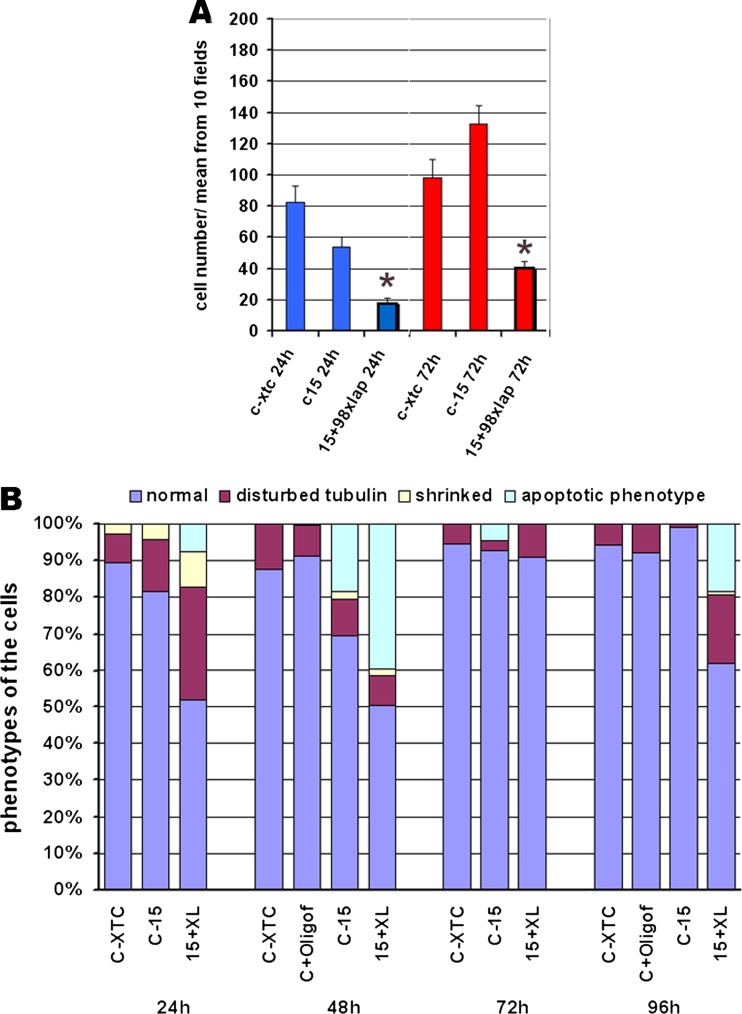
Microscopic immunofluorescence analyses of XTC cells subjected to knockdown or mock-knockdown procedures. Knockdown of XLAP2 protein in XTC cells results in inhibition of growth, increased cell death and cellular abnormalities. **a**, **b** Tissue cultured cells were grown on glass coverslips and transfected either with 15XLAP2 and 98XLAP2 siRNAs or scrambled control siRNA (C-15). Untreated cells (C-XTC) were used as a control. After the indicated period of time, cells were fixed and costained for XLAP2 and α-tubulin and DNA was visualized with DAPI (not shown). **a** Cells from 10 representative fields were counted. The diagram represents total cell count from 10 fields. *Asterisk* denotes statistically significant lower cell number in 15 + 98xlap siRNA treated XTC cells versus c15 siRNA control cells (*P* < 0.05). **b** Analysis of cellular organization in XTC cells from 10 fields. Normal, well-organized tubular network, average nucleus size; disturbed tubulin, cells showing partially condensed tubulin but attached to the surface, small nucleus; shrinked, condensed tubulin, small nucleus; apoptotic phenotype, weak or no tubulin staining, no NE staining, degraded DNA. XLAP2 knockdown results in phenotype abnormalities in XTC cells, as well as in cell death

Subsequent large-scale analyses of the siRNA transfected cells revealed the toxic effect of the XLAP2 protein knockdown and a drastically decreased growth of XTC cultures. Figure 4 demonstrates the representative results of one series of such experiments. When cell number was analyzed (Fig. 4a), we clearly observed the toxic effect of XLAP2 knockdown on XTC cells after 24 and 72 h comparing to control cells and control siRNA treatment. The analyses of the cell phenotypes after XLAP2 knockdown also demonstrated increased percentage of the cell shape abnormalities (Fig. 4b). Cells treated with siRNA had increased a percentage of apoptotic phenotype, were shrinked, and/or had disturbed microtubule network.

Based on the analyses of cells still attached to cover glass at the time of observation (Fig. 4), the first visible effect to appear (after 24 h) is the disturbed cytoskeleton and shrinking of the cells with only a fraction of cells with apoptotic phenotype.

At 48 h, almost exactly the same number of cells showed abnormal phenotype, but most of them were apoptotic. Similar temporal effect of the appearance of abnormal and apoptotic phenotype is observed in the cycle between 72 and 96 h (Fig. 4) but obscured by proliferating normal (non-transfected) cells (Fig. 4a).

Based on the fact that we have detected only a few mitotic cells with LAP2 knockdown, we may conclude that, in general, knockdown of XLAP2β in XTC cells either stops the cells from entry into mitosis or cells detach themselves from the surface of culture vessel/cover glass before mitotic entry or both. We propose that this effect is the result of abnormal nucleus re-assembly when cells lack XLAP2β protein.

### XLAP2β knockdown affect cell nuclei morphology and location of lamin B2 and nucleoporins

Xenopus XTC cells are not easy to transfect; therefore, in most of our experiments, we could not get sufficient efficiency of transfection for detailed studies of cell cycle or pathways affected using bulk tissue culture transfection. Due to the toxicity of XLAP2β knockdown, we were unable to select stable cell line with constitutive protein knockdown as well.

**Fig. 5:**
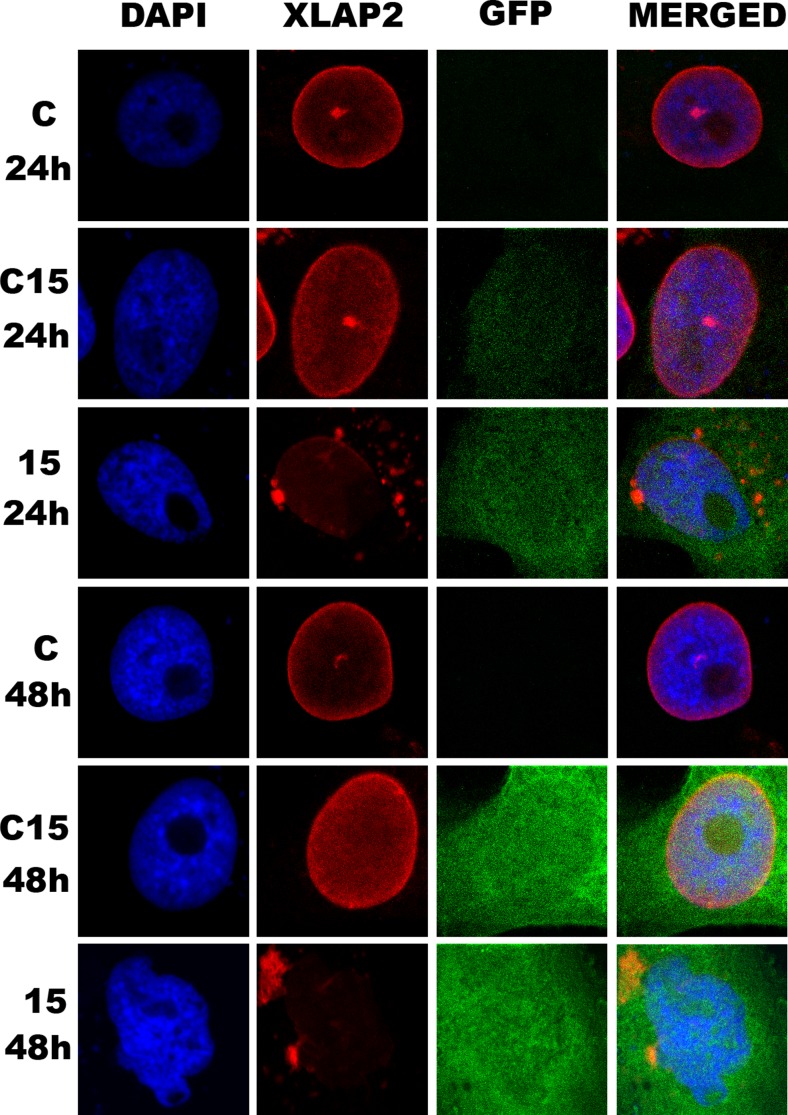
Knockdown of XLAP2β in tissue cultured *X. laevis* XTC cells results in cell abnormalities. XTC cells were transfected with plasmid encoding antisense (15) or scrambled (C15) siRNA together with GFP as a transfection marker directed against an N-terminal fragment of XLAP2. For immunofluorescence, cells were grown on glass coverslips for 24 or 48 h after transfection, fixed in PFA, and stained for XLAP2 (*red*) and DNA (DAPI, *blue*), GFP (*green*). Single confocal sections (1.5 μm) through the center of nuclei are shown. Bar: 5 μm. Untreated cells (*C*) or cells treated with a plasmid encoding scrambled siRNA and GFP (C15) were used as controls. Note that nuclear shape abnormalities are concomitant with loss of XLAP2 protein from NE. Note XLAP2 speckles and discontinuous XLAP2 in NE in nucleus transfected with 15 siRNA plasmid after 24 h and lobular shape of apoptotic nucleus after 48 h

Therefore, in order to perform knockdown experiments in more controllable system allowing for easy detection of transfected cells under microscope, presumably with decreased level of XLAP2β protein, we switched to plasmid-based siRNA transfection system (pFIV-H1/U6-copGFP) with GFP (green fluorescent protein) reporter protein as a marker for transfected cells. Plasmid encoded siRNA system due to the GFP reporter protein expression makes available the distinction of the particular transfected cell for study. Additionally, when possible, the level and distribution of XLAP2 protein were visualized. Thus, we were able to monitor the knockdown effect in transfected cells only (Fig. 5) and monitor the level and location of XLAP2β protein together with other proteins in multichannel confocal microscopy.

Data indicated that although control siRNA has some minor effect on cell morphology, and XLAP2β protein was distributed in more diffused (partly nucleoplasmic) pattern than in non-transfected cells, only antisense siRNA was able to knockdown XLAP2 protein expression. This correlated with the abnormal nuclear shape and atypical chromatin staining (Fig. 5).

Statistical analyses of cells with GFP for expression of XLAP2β protein using confocal microscopy demonstrated that there is a very good correlation between GFP expression and decreased level of XLAP2β in cells transfected with the silencing construct. Eighty-six percent of cells expressing GFP showed decreased level of XLAP2β, and this downregulation, in 80 % of cases, was associated with the abnormal phenotype of nuclei (Additional file 3: Figure S3).

Typical nuclear abnormalities most frequently observed in cells with decreased level of XLAP2β protein were demonstrated in details in Additional file 4: Figure S4. The cells with decreased level of LAP2β protein exhibited changed shape of cell nuclei and FG-repeat nucleoporins relocation into the nucleoplasm. Nuclear pore complexes (NPCs) form clusters, frequently elongated or forming lines (Additional file 4: Figure S4). Cells with low level of XLAP2β also showed discontinuous rim-like staining of the NE and NL and had typically abnormal shape and chromatin distribution similar to those observed in cells from EDMD patients (laminopathy of a muscular dystrophy type) (Fig. 5 and Additional file 4: Figure S4). During analyses of hundreds of XTC cells using this knockdown system, we also did not observe mitotic cells with decreased level of LAP2β protein.

**Fig. 6:**
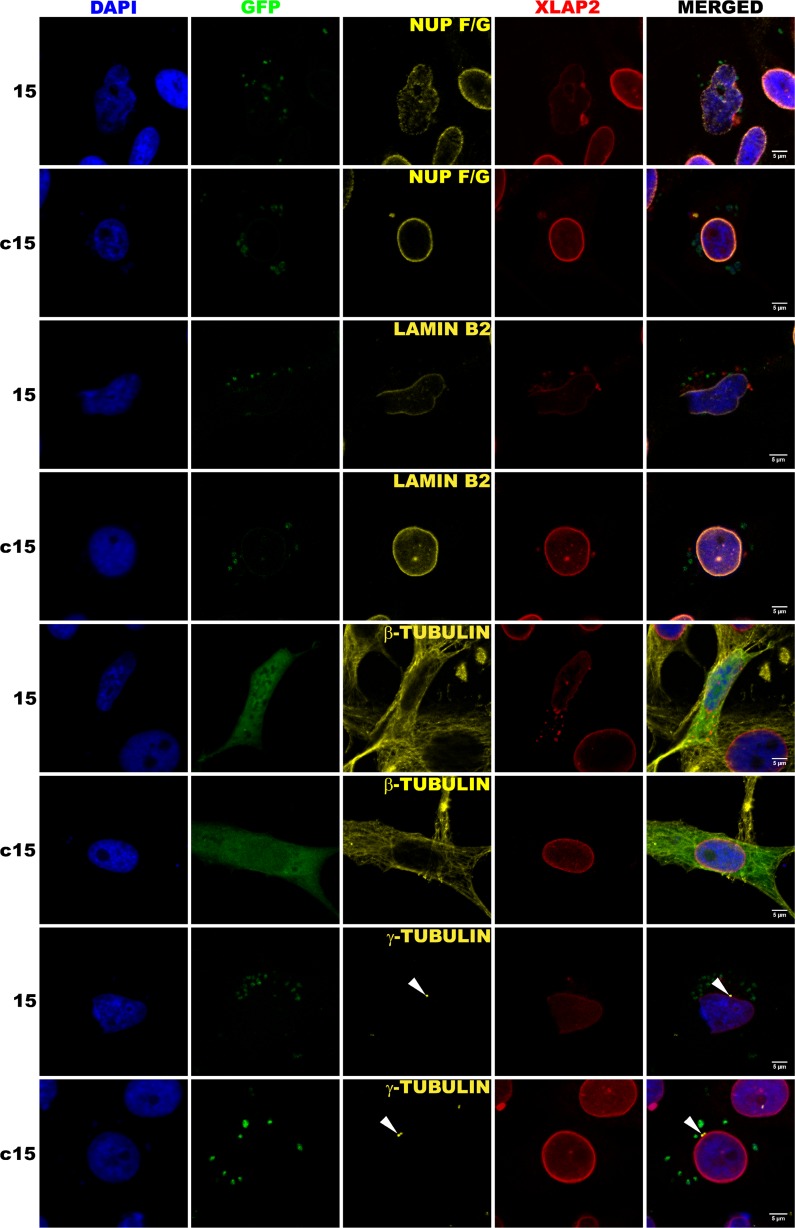
XLAP2β knockdown affects cell nuclei morphology and interferes with the proper location of lamin B2 and nucleoporins. XTC cells were transfected with siRNA plasmid, encoding silencing (15) or scrambled (C15) siRNA with GFP as a transfection marker, directed against an N-terminal fragment of XLAP2. For immunofluorescence, cells were grown on coverslips for 48 h after transfection, fixed with PFA or methanol, and stained for XLAP2β (*red*), DNA (*blue*), and nucleoporins with F/G repeats, lamin B2, β- and γ-tubulin (*yellow*). Expressed GFP proteins (*green*) localize throughout the whole cell volume. For most images, GFP looks precipitated and weakly visible because of methanol fixation method used to obtain the best performance of individual antibodies. Single confocal sections (1.5 μm) through the center of nuclei are shown. Bar: 5 μm. XTC cells with decreased level of XLAP2β protein have abnormally shaped nuclei, and F/G-repeat nucleoporins and lamin B2 are redistributed. The microtubule network is frequently shrunk, but centrosomes remain their normal position directly at the nuclei

Detailed analyses of the effect of XLAP2β protein knockdown on the distribution of selected proteins during interphase are demonstrated in Figure 6. It is shown that in XTC cells with decreased level of XLAP2β protein, both FG-repeat nucleoporins and lamin B2 protein were redistributed. In case of FG-repeat nucleoporins, changes in their distribution consist in the loss of its regular rim-like pattern in favor of line-like or granular-like pattern with proteins diffused throughout the nucleoplasm. LAP2β knockdown resulted in abnormal location of lamin B2. Typically, lamin B2 and NPCs were absent from peripheral region of one side of cell nucleus (Additional file 4: Figure S4). Microtubule network was also frequently affected, but we did not find any statistical correlation between XLAP2β knockdown and location and distance of centrosomes to the NE.

## Discussion

### XLAP2 distribution in XTC cells and question of TPX2

Our data demonstrated that XLAP2β protein localizes mostly to the NE of XTC cells during interphase. During this phase, XLAP2 colocalizes with typical NE and NL antigens as lamin B2 (Fig. 1a). Apparent colocalization with nucleoporins (Fig. 1a) does not indicate their interaction. Higher resolution images (STED) or SEM/TEM studies demonstrated that they do not colocalize (Chmielewska et al. 2011). There is no significant colocalization between most of XLAP2 and TPX2 protein during interphase (Fig. 2) particularly at the nuclear envelope. Also, TPX2 protein does not interact with XLAP2 protein as revealed by our co-IP experiments (Additional file 2: Figure S2) on XTC cells. This documents that XLAP2β protein does not colocalize nor interact with TPX2 protein during interphase as XLAP2ω and γ isoforms do in frog egg extract (O'Brien and Wiese 2006). This suggests that XLAP2β isoform shows different properties in this respect than isoforms γ and ω which are present in mature *X. laevis* eggs (Chmielewska et al. 2011). This can be simply accounted for different exon composition between β and ω and γ isoforms.

### Knockdown of XLAP2 and cell death mechanisms

XTC cells, besides A6 cells, are the only well characterized *X. laevis* cells available for studies in tissue cultured cells model. Both cell types are difficult to transfect, but XTC cells seem to offer a small advantage of giving higher efficiency of transfection (up to 50 % typically). This makes impossible to perform whole cell culture studies (Western blot analyses of signaling pathways, etc.). Our attempts to isolate transfected or transduced cells using FACS and GFP reporter protein, using pFIV-H1/U6-copGFP based constructs and virus, failed because the expression of reporter protein was simultaneous with expression of siRNA that was toxic and resulted in detachment of cells with decreased XLAP2 protein synthesis. Subsequently, our attempt to select stable cell lines with inducible expression of siRNA failed probably due to the inefficient switching off of the promoter for siRNA. Thus, our experimental procedures were limited to confocal microscopy analyses of cells with reporter protein–GFP marking transfected cells. There was a very good correlation between GFP expression and knockdown of XLAP2 protein (Additional file 3: Figure S3), so we adopted this method for all subsequent studies.

We observed that cells with decreasing levels of XLAP2β protein gradually accumulated abnormalities in size, shape, and microtubule network (Fig. 4, Additional file 4: Figure S4), leading finally to cell detachment from the surface. These changes were accompanied by changes in chromatin and nuclear morphology, NE structure, and distribution of nuclear and NPC proteins (Figs. 5 and 6). A surprising feature of cells with a low level of XLAP2β was that we could not detect mitotic cells. This suggests that lack of LAP2β prevents cells from mitotic entry or that abnormally re-assembled nuclei (and chromatin) in such cells resulted in cell cycle block at G0/G1 or G1/S/G2. The major effect of XLAP2β knockdown was structural abnormalities of the cells, which were followed by appearance of apoptotic phenotype. This would favor the hypothesis that apoptosis is a secondary effect of XLAP2β knockdown in XTC cells.

Since isoform β is the only LAP2 protein in XTC cells, this may account for the severity of knockdown phenotype though there may be several plausible mechanisms involved. In *X. laevis*, at least three different isoforms were detected during development (Chmielewska et al. 2011). Since they share several common domains, some of the functions may be conserved between them. Domains with no similarity between them may be responsible for different properties, unique for particular isoform. XLAP2β was found to be associated with clusters of heterochromatin both next to NE and internally in cell nuclei. As we have previously shown, XLAP2β was present inside the cell nucleus both in nuclear envelope invaginations and without visible nuclear membranes in interphase cells (Chmielewska et al. 2011). Since mammalian LAP2β interacts with HDAC3 (Somech et al. 2005), this protein may be anchored on heterochromatin by Xenopus LAP2β protein and, thus, maintain the inactive status of heterochromatin. The knockdown of XLAP2β may result in the release of HDAC3 from heterochromatin loci and change in location and transcription activity, or both. Similar results were shown for lamin B receptor (LBR), inner nuclear membrane protein responsible for heterochromatin positioning at nuclear periphery, present in NE, and membrane invaginations inside the cell nucleus (Ellenberg et al. 1997). Knockdown of LBR by morpholino antisense nucleotides in 1–2 cell zebrafish embryos caused reduced viability and severe morphological alterations in surviving embryos (Schild-Prufert et al. 2006). RNAi-mediated knockdown of LBR in HeLa cells resulted in failure of NE reassembly and abnormal chromatin decondensation after mitosis leading to the apoptotic cell death (Lu et al. 2010). On the other hand, disruption of LBR function in differentiating cells was responsible for the loss of peripheral heterochromatin and inverted nuclear morphology with heterochromatin present in nuclear interior, which in turn altered gene expression profile (Solovei et al. 2013).

Mechanism that may function during mitosis is associated with the structural role of LAP2β protein in the maintenance of nuclear structure and reassembly during mitosis. The lack of XLAP2β – LEM domain integral membrane of INM and loss of one of the links between chromatin and NE (chromatin-BAF-LAP2β at NE) may result in abnormal nucleus and NE reassembly observed in our studies (Figs. 5 and 6). Since in Xenopus cells, many different LEM domain proteins are expressed besides LAP2β, the severity of phenotype is lower than LBR knockdown.

Proper assembly of lamin B and nucleoporins is necessary for DNA replication, transcription, and nuclear pore spacing. Defects in their distribution affect all these processes (Smythe et al. 2000, Moir et al. 2000, Spann et al. 2002, Margalit et al. 2005). XLAP2β knockdown disrupts lamin B2 and NPC protein location at NE and NL, which in turn can affect these processes. On top of the above mechanisms lack of LAP2β leads to the redistribution of lamin B2 and probably the relocation of lamin A since both proteins interact with LAP2 proteins. This may further enhance the above mechanisms that finally results in the cells death after XLAP2β expression knockdown.

## Additional files

Below is the link to the electronic supplementary material.

Additional file 1 Figure S1 XLAP2β and TPX2 show different subcellular distribution pattern in XTC interphase cells. Cells were grown on coverslips, fixed with PFA or methanol and stained for TPX2, lamin B2, nucleoporins F/G, β-tubulin, and membranes. DNA was visualized with DAPI (blue). Single confocal sections (1,5 μm) through the center of nuclei are shown. Scale is as indicated in the picture. Detailed analysis of TPX2 distribution in interphase cells along with the NE antigens, which are known to colocalize with XLAP2β, revealed that TPX2 has different distribution pattern from NE proteins: laminB2 or Nup F/G (Figure S1A) and XLAP2β too (for comparison see at Fig. 1). TPX2 does not localize to the cytoplasm or MTOC (Figure S1A). Overall TPX2 expression level analysis in interphase XTC cells demonstrated that only 40 % of cells have detectable TPX2 level (using IF). This fraction of cells was evaluated as a mid-S phase to M phase cells (Figure S1B). (TIFF 1272 kb)

Additional file 2 Figure S2. Co-immunoprecipitation followed by MS/MS strongly suggests there is no interaction of XLAP2 and TPX2 in interphase XTC cells. Potential interactions between XLAP2 and TPX2 proteins were analyzed using immunoprecipitation for XLAP2 followed by tandem mass spectrometry for identification of co-immunoprecipitated proteins from unsynchronized XTC cells. Figure S2A shows western blot from immunoprecipitation experiments with anti-XLAP2 Igs (IP1 – IP3 lanes) and control Igs stained with anti-XLAP2 antibodies as a control for XLAP2 presence in all IP samples. *IP1, IP2* and *IP3* – resulted co-immunoprecipitated proteins from unsynchronized XTC cells extracts in different ionic conditions (more details in *Materials and Methods* section), control- Immuno- and co-immunoprecipitated protein’s eluates from unsynchronized XTC cells extracts, *Load* – starting XTC cells extract, *IgH* – immunoglobulins’ heavy chain. Figure S2B displays MS/MS results analyzed by Mascot and Scaffold3 software. Any of IPs performed with anti-XLAP2 Igs revealed no TPX2 protein that indicates no detectable interactions between both proteins are experimental conditions. Please note that lamin A protein (together with many other proteins) is present in co-IP experiments. *1, 2* and *3* in the table’s headline correspond to *IP1, IP2* and *IP3*, respectively and *C* is the same as control, % - Protein Identification Probability. (TIFF 525 kb)

Additional file 3 Figure S3. There is high correlation between GFP expression and decreased level of XLAP2. XTC cells were prepared like for Fig. 6. and stained for XLAP2β and DNA. Only cells expressing GFP were counted, with simultaneous estimation of XLAP2β level and nuclei conditions. Detailed statistical analysis of cells after transfection with plasmid-based XLAP2β siRNA revealed that 86 % of cells with GFP exhibit decreased a level of XLAP2β. 80 % of those cells show nuclei abnormalities. Transfection with scrambled siRNA plasmid gave rise for such phenotypes in reverse proportions. Only 20 % of the cells appear to have an altered level of XLAPβ protein. (TIFF 154 kb)

Additional file 4 Figure S4. The effect of the XLAP2 knockdown in XTC cells. Cells were prepared as in Fig. 3. and stained for XLAP2β (red), nucleoporins m414 (yellow, first and second row), lamin B2 (yellow, third row) and β-tubulin (yellow, fourth row). DNA was visualized with DAPI. Bar: 5 μm. Co-staining for XLAP2 and other antigens reveals loss of XLAP2 protein. XLAP2 knockdown in XTC cells transfected with plasmid encoding antisense siRNA results in nucleus abnormalities which is irregular and aberrant in shape, abnormal chromatin distribution, partial loss of NE, mislocalization and dispersion of nucleoporin m414 in “granular pattern” through entire nucleus and do not affect MTOC position which is located typically next to NE. (TIFF 1851 kb)
