# Supplementary figures and images for: Xenopus LAP2β protein knockdown affects location of lamin B and nucleoporins and has effect on assembly of cell nucleus and cell viability
Source: Protoplasma. 2015 Jul 25;253(3):943–56. doi: 10.1007/s00709-015-0861-y (PMC4819936; doi:10.1007/s00709-015-0861-y)

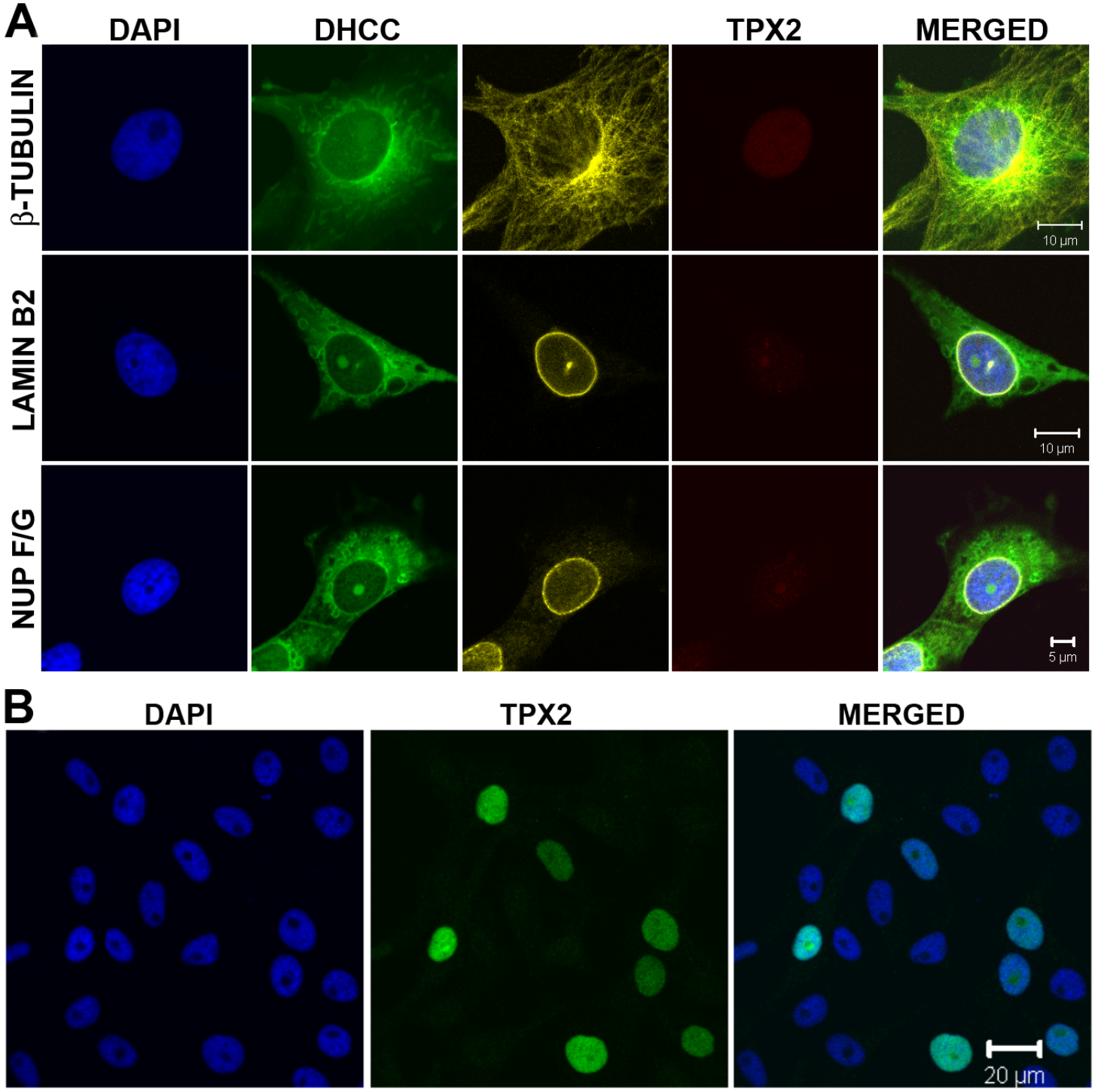

Supplement: Supplementary file 1 — Figure S1 XLAP2β and TPX2 show different subcellular distribution pattern in XTC interphase cells. Cells were grown on coverslips, fixed with PFA or methanol and stained for TPX2, lamin B2, nucleoporins F/G, β-tubulin, and membranes. DNA was visualized with DAPI (blue). Single confocal sections (1,5 μm) through the center of nuclei are shown. Scale is as indicated in the picture. Detailed analysis of TPX2 distribution in interphase cells along with the NE antigens, which are known to colocalize with XLAP2β, revealed that TPX2 has different distribution pattern from NE proteins: laminB2 or Nup F/G (Figure S1A) and XLAP2β too (for comparison see at Fig. 1). TPX2 does not localize to the cytoplasm or MTOC (Figure S1A). Overall TPX2 expression level analysis in interphase XTC cells demonstrated that only 40 % of cells have detectable TPX2 level (using IF). This fraction of cells was evaluated as a mid-S phase to M phase cells (Figure S1B). (TIFF 1272 kb) [file 709_2015_861_MOESM1_ESM.tif]

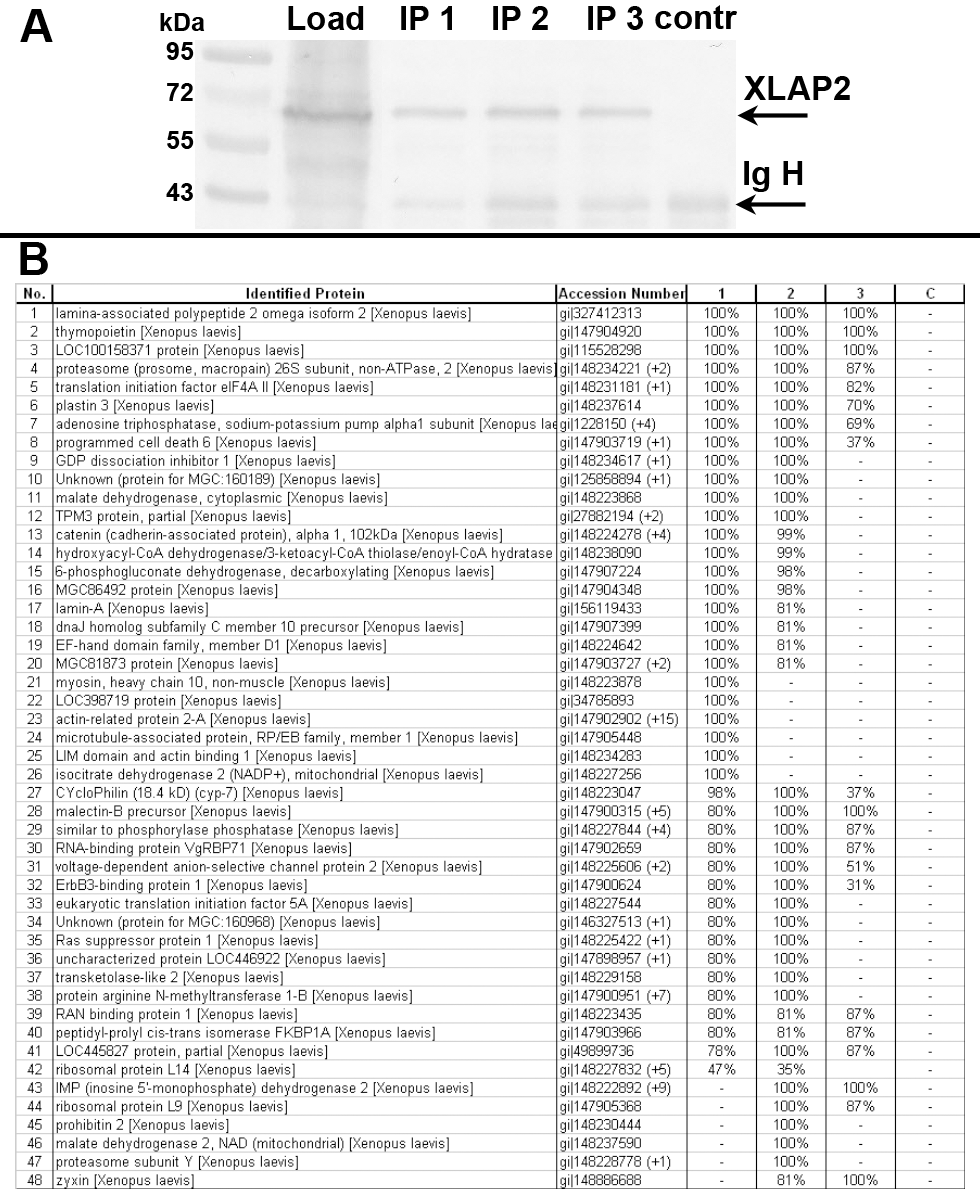

Supplement: Supplementary file 2 — Figure S2. Co-immunoprecipitation followed by MS/MS strongly suggests there is no interaction of XLAP2 and TPX2 in interphase XTC cells. Potential interactions between XLAP2 and TPX2 proteins were analyzed using immunoprecipitation for XLAP2 followed by tandem mass spectrometry for identification of co-immunoprecipitated proteins from unsynchronized XTC cells. Figure S2A shows western blot from immunoprecipitation experiments with anti-XLAP2 Igs (IP1 – IP3 lanes) and control Igs stained with anti-XLAP2 antibodies as a control for XLAP2 presence in all IP samples. IP1, IP2 and IP3 – resulted co-immunoprecipitated proteins from unsynchronized XTC cells extracts in different ionic conditions (more details in Materials and Methods section), control- Immuno- and co-immunoprecipitated protein’s eluates from unsynchronized XTC cells extracts, Load – starting XTC cells extract, IgH – immunoglobulins’ heavy chain. Figure S2B displays MS/MS results analyzed by Mascot and Scaffold3 software. Any of IPs performed with anti-XLAP2 Igs revealed no TPX2 protein that indicates no detectable interactions between both proteins are experimental conditions. Please note that lamin A protein (together with many other proteins) is present in co-IP experiments. 1, 2 and 3 in the table’s headline correspond to IP1, IP2 and IP3, respectively and C is the same as control, % - Protein Identification Probability. (TIFF 525 kb) [file 709_2015_861_MOESM2_ESM.tif]

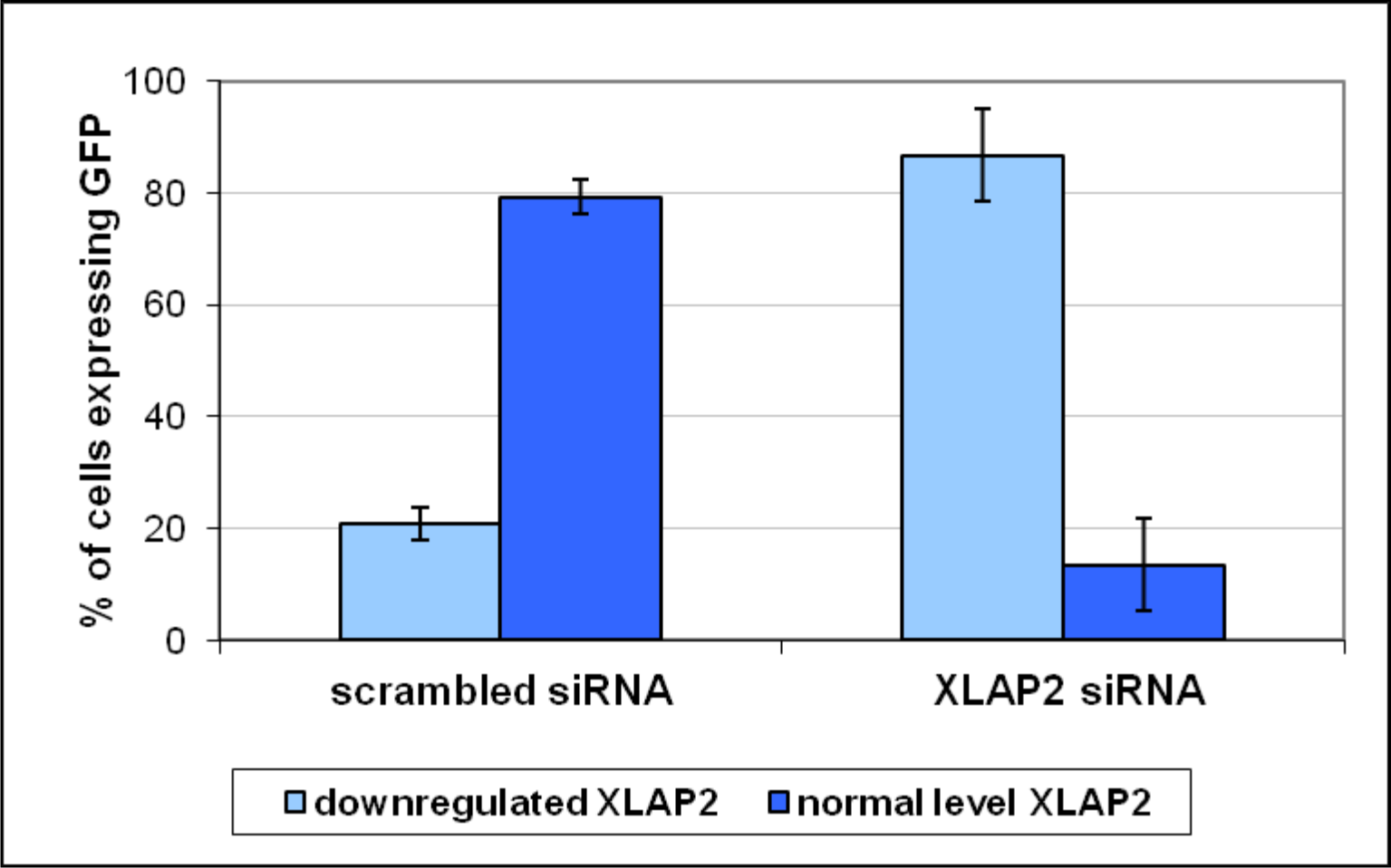

Supplement: Supplementary file 3 — Figure S3. There is high correlation between GFP expression and decreased level of XLAP2. XTC cells were prepared like for Fig. 6. and stained for XLAP2β and DNA. Only cells expressing GFP were counted, with simultaneous estimation of XLAP2β level and nuclei conditions. Detailed statistical analysis of cells after transfection with plasmid-based XLAP2β siRNA revealed that 86 % of cells with GFP exhibit decreased a level of XLAP2β. 80 % of those cells show nuclei abnormalities. Transfection with scrambled siRNA plasmid gave rise for such phenotypes in reverse proportions. Only 20 % of the cells appear to have an altered level of XLAPβ protein. (TIFF 154 kb) [file 709_2015_861_MOESM3_ESM.tif]

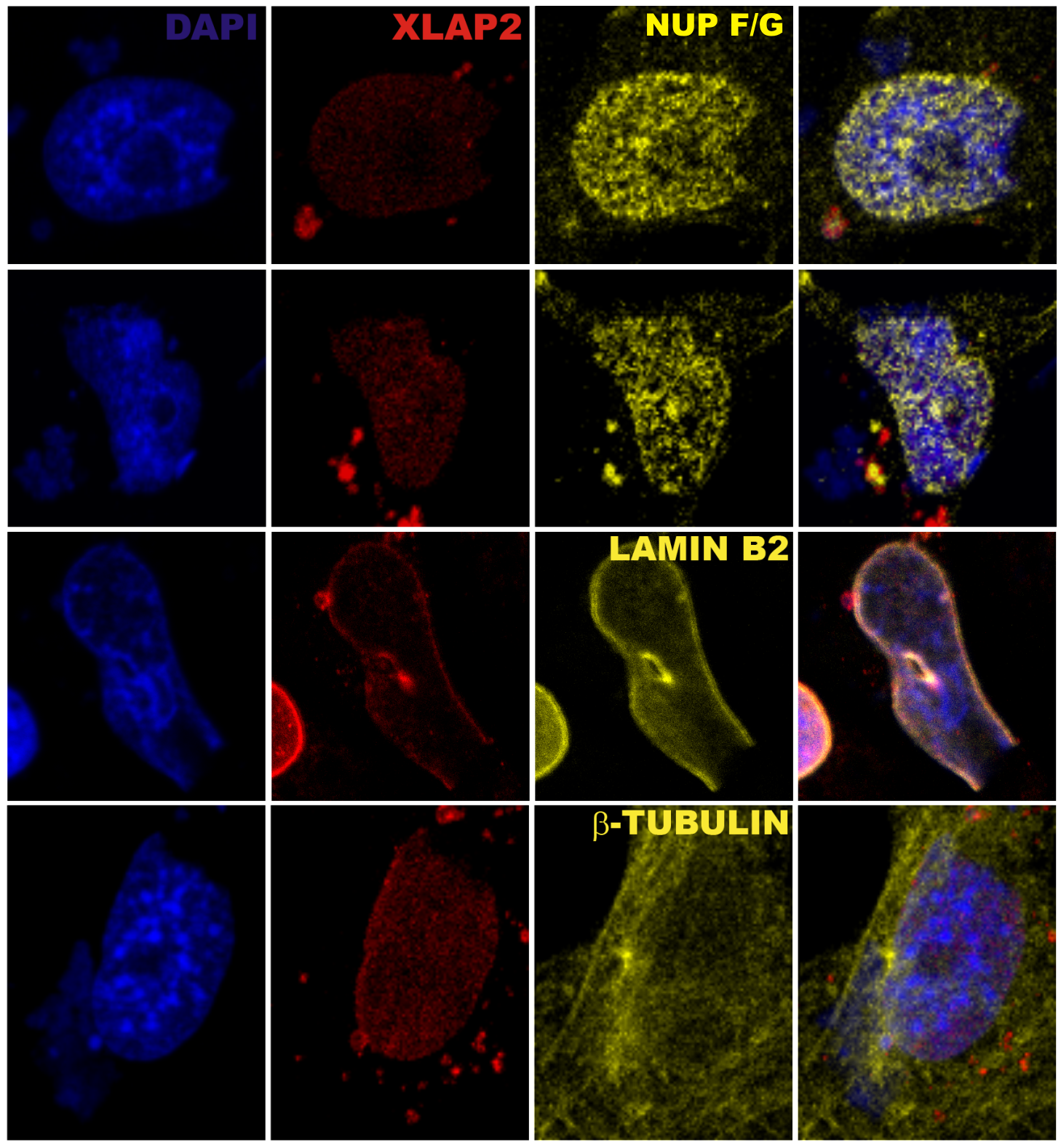

Supplement: Supplementary file 4 — Figure S4. The effect of the XLAP2 knockdown in XTC cells. Cells were prepared as in Fig. 3. and stained for XLAP2β (red), nucleoporins m414 (yellow, first and second row), lamin B2 (yellow, third row) and β-tubulin (yellow, fourth row). DNA was visualized with DAPI. Bar: 5 μm. Co-staining for XLAP2 and other antigens reveals loss of XLAP2 protein. XLAP2 knockdown in XTC cells transfected with plasmid encoding antisense siRNA results in nucleus abnormalities which is irregular and aberrant in shape, abnormal chromatin distribution, partial loss of NE, mislocalization and dispersion of nucleoporin m414 in “granular pattern” through entire nucleus and do not affect MTOC position which is located typically next to NE. (TIFF 1851 kb) [file 709_2015_861_MOESM4_ESM.tif]
